# Dispersion of *Lutzomyia longipalpis* and expansion of visceral leishmaniasis in São Paulo State, Brazil: identification of associated factors through survival analysis

**DOI:** 10.1186/s13071-018-3084-1

**Published:** 2018-09-10

**Authors:** Agda M. Oliveira, Rossana V. M. López, Margareth R. Dibo, Lilian A. C. Rodas, Marluci M. Guirado, Francisco Chiaravalloti-Neto

**Affiliations:** 1 Department of Epidemiology, Faculdade de Saúde Pública da Universidade de São Paulo, Sao Paulo, Sao Paulo Brazil; 2 Center for Translational Research in Oncology, Instituto do Câncer do Estado de São Paulo, Sao Paulo, Sao Paulo Brazil; 3 Laboratory of Biochemistry and Molecular Biology, Superintendência de Controle de Endemias, Sao Paulo, Sao Paulo Brazil; 4 Regional Service 9. Superintendência de Controle de Endemias, Araçatuba, Sao Paulo Brazil; 5 Laboratory of Vectors of São José do Rio Preto, Superintendência de Controle de Endemias, São José do Rio Preto, Sao Paulo Brazil

**Keywords:** Survival analysis, Visceral leishmaniasis, Neglected tropical disease, Brazil

## Abstract

**Background:**

In Brazil, visceral leishmaniasis (VL) is a serious public health problem because of its magnitude, geographical expansion and potential harms caused by illnesses, including death. However, VL is largely ignored in discussions of tropical disease priorities. Thus, this study aimed to identify factors associated with the expansion of VL and the dispersion of its vector, *Lutzomyia longipalpis*, in the municipalities of the State of São Paulo, Brazil.

**Methods:**

Information about the date of vector detection and the confirmation of autochthonous VL occurrence in humans and canines in São Paulo were obtained between 1997 and 2014. Survival curves were calculated by the Kaplan-Meier and the Cox multiple regression models was used.

**Results:**

The presence of the Marechal Rondon highway showed the highest positive association with vector dispersion and canine and human VL expansion. The monthly maximum and minimum temperature averages recorded in the municipalities during the study period were also positively associated with these events. The presence of transverse highways was positively associated with the presence of the vector; the border with the State of Mato Grosso do Sul, the presence of a prison, microregion headquarters, and the presence of the Tietê River were positively associated with the occurrence of canine cases, while only the presence of prison was positively associated with the occurrence of human cases. The construction of the Bolivia-Brazil gas pipeline was not associated with any events.

**Conclusions:**

Survival analysis enabled the identification of factors associated with vector dispersion and VL expansion, thus the results of this study may be useful to the improvement of VL surveillance and control activities in the State of São Paulo and throughout Brazil.

**Electronic supplementary material:**

The online version of this article (10.1186/s13071-018-3084-1) contains supplementary material, which is available to authorized users.

## Background

Leishmaniasis is one of the most neglected tropical diseases that affects people worldwide, and visceral leishmaniasis (VL), one of its forms, can cause mortality when not properly treated [1]. Yet, even with treatment access, 10–20% of VL cases result in death [2]. Globally, an estimated 200,000–400,000 new cases of VL occur per year, with more than 90% of the cases concentrated in India, Nepal, Sudan, Bangladesh and Brazil [3, 4]. In Latin America, VL has been reported in 12 countries and Brazil has been responsible for approximately 96% of the reported cases [5]. In Brazil, VL is a serious public health problem because of its magnitude, geographical expansion and potential to cause serious health illnesses. Initially, the occurrence of VL was restricted to rural areas, but the adaptation of its main vector, *Lutzomyia longipalpis* (Lutz & Neiva, 1912), to anthropogenic environments allowed VL to reach urban areas of Brazilian cities [6]. In addition, the main reservoir of VL is the domestic dog, which facilitates its dispersion through these urban areas. Despite these known issues, VL is largely ignored in discussions of tropical disease priorities [7, 8].

Authors have associated the dispersion of the vector and the geographical expansion of VL with the destruction or modification of natural environments, irregular occupation of areas, lack of basic sanitation, poverty, big constructions and migratory flow, among other factors [6, 9–15]. For example, studies in the states of Mato Grosso do Sul [11] and São Paulo (SP) [16] indicated the role of the BR-262 and Marechal Rondon highways (the first one crosses the state of Mato Grosso do Sul from the Bolivian border to the SP border and the second one is its continuation in SP) and the construction of the Bolivia-Brazil gas pipeline in the expansion of human VL in Brazil. The association of human VL and the gas pipeline is presumed to be related to the movement and migration of a large number of construction workers [11], while the association with large highways is presumed to be related to their construction and expansion, facilitating the movement and migration of people and animals, including those affected by canine or human VL [9, 11, 13–17]. Oliveira et al. [18] showed that the dispersion of *Lu. longipalpis* in the northwest region of SP has been through neighborhoods of the municipalities and highlighted the role of the roads between them.

In 1997, the first record of a vector presence in a SP urban area was in Araçatuba [19], which was followed by the first report of an autochthonous case of canine VL in 1998 and an autochthonous case of human VL in 1999 in the same city. From Araçatuba, vector and VL distribution expanded along a main axis in the northwest-southeast direction and, from here, the distribution expanded in north and south directions [10, 16, 20].

Other factors related to migratory flow that could be related to the dispersion of the vector and the expansion of the disease in SP are the presence of the Euclides da Cunha highway [18], proximity to the Mato Grosso do Sul and Minas Gerais currencies [10, 16, 21, 22], presence of the Tietê River [23] and presence of prisons; however, there is no known association between the presence of prisons and VL dispersion and occurrence in the literature. In the current context of Brazilian prisons, the institutions have been identified as triggers of population movement and migration [24, 25]. The connection of the Tietê River to the dispersion and occurrence of VL could be related to the fact that it is a waterway and natural recreational area, which facilitates the movement of people.

In addition, climatic conditions must be considered, as these factors would have a major influence on *Lu. longipalpis* and, consequently, on the occurrence of disease in dogs and humans. Several studies have reported an association between temperature, humidity, and precipitation and vector distribution [14, 15, 26–28], as well as with the distribution of canine and human VL [15].

To better understand the dynamics and underlying mechanisms of *Lu. longipalpis* dispersion and VL expansion in SP, this study aimed to describe these phenomena and evaluate their relationship with associated factors, especially those that have been cited in the literature. The clarification of these relationships has the potential to improve the surveillance and control activities of VL, both in SP and in other regions of the country.

## Methods

### Study design and area

**Fig. 1:**
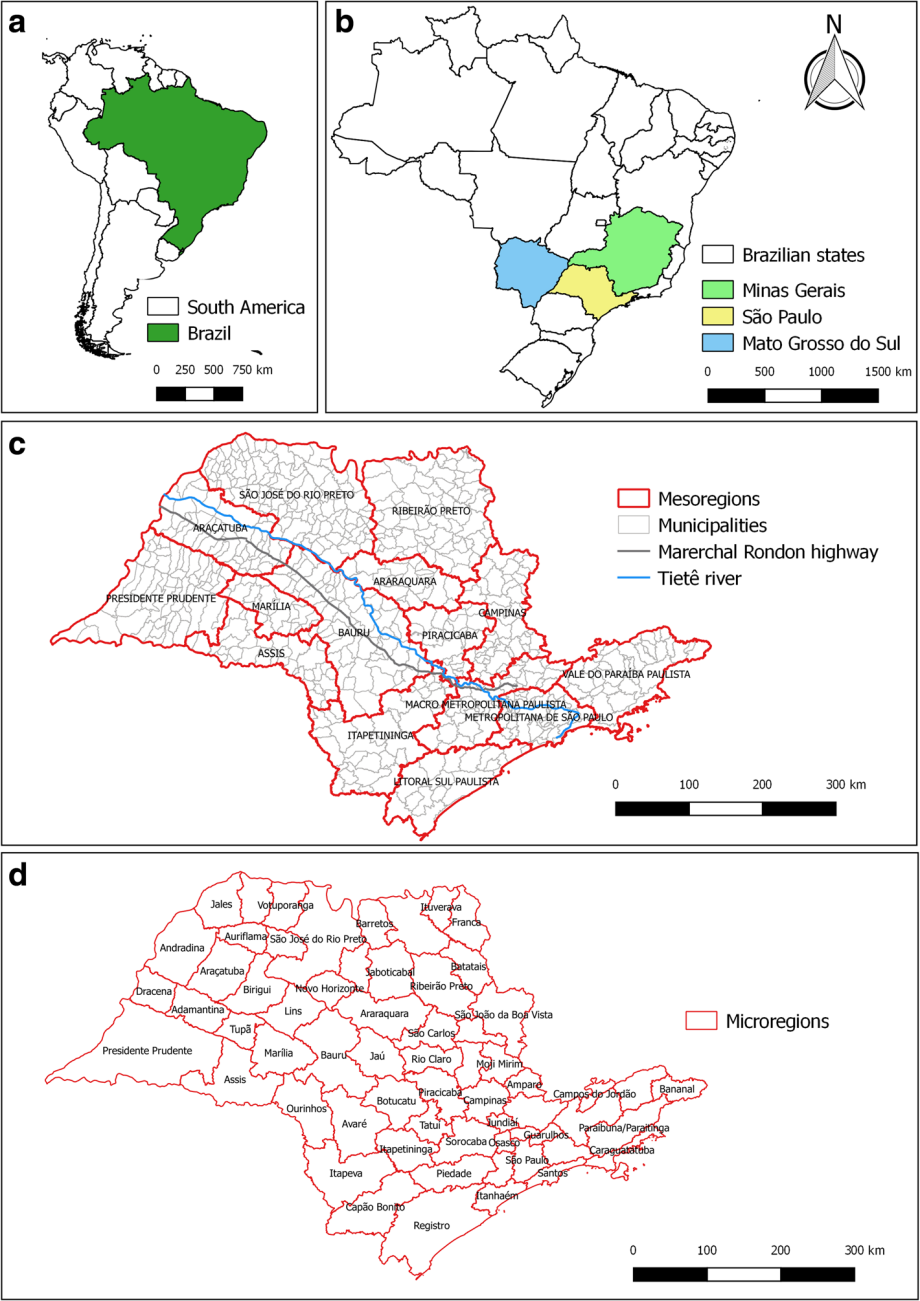
Location of the states of São Paulo (study area), Minas Gerais and Mato Grosso do Sul in Brazil and South America (**a** and **b**); mesoregions, municipalities, the Marechal Rondon Highway, and the Tietê River in the State of SP, with an emphasis on Araçatuba, the municipality to first report the detection of *Lu. longipalpis* and presence of VL cases in SP (**c**); microregions of the State of SP (**d**)

We developed a longitudinal study using survival analysis. The study area compromised 645 municipalities in the State of SP, with a population of 41,252,160 inhabitants, as of 2010, that were grouped into 15 mesoregions and 63 microregions (Fig. 1) [29]. Despite our information being groupings (the municipalities), they were treated as individuals, justifying the choice of a longitudinal study.

### Data source and variables

Secondary data from VL cases in humans and autochthonous canines as and on the presence of the vector between 1997 and 2014 were obtained from two organizations belonging to the State Department of Health of the State of São Paulo: the Superintendency of Control of Endemics and the Center for Epidemiological Surveillance Professor Alexandre Vranjac. Information regarding the six municipalities that reported the presence of the vector prior to this period was obtained from the literature [10, 20, 30, 31]. Based on these data sources, the following dependent variables were obtained, with values for each of the 645 municipalities: dates (month and year) of registration of the presence of the vector, the first registration of a canine autochthonous case and human case. For the six municipalities with vector detection prior to 1997, the beginning of the study period was adopted as the detection date. If the month of vector detection and confirmation of a canine or autochthonous human case was not available, these events were considered to occur in the middle of the year (June). The present study did not include maps of vector dispersion or of the expansion of VL canine and human cases because Casanova et al. [10] previously reported and illustrated both of these maps, as well as a map of the expansion of the arthropod vector in SP.

In the present study, the independent variables, for which values were obtained from each of the 645 municipalities of SP, were considered to be the following: the microregion headquarters; the presence of the Marechal Rondon and Euclides da Cunha highways (radial highways); the presence of transverse highways; the presence of the Tietê River, the border of the states of Mato Grosso do Sul and Minas Gerais; the presence of a prison; the Municipal Human Development Index (MHDI); and the simultaneous or non-simultaneous occurrence of the construction of the Bolivia-Brazil gas pipeline in relation to the presence of the vector and the disease in dogs and humans. This last covariable was considered as a simultaneous occurrence if the detection of the vector occurred during the construction period or within one year of its completion in a given municipality. The same criterion was used to define the simultaneous occurrence of the construction and the detection of the first canine case and the first autochthonous human case. Regarding the classification of the highways, a radial highway was considered to be a route that connected the municipalities in the direction of the capital (the Marechal Rondon and Euclides da Cunha highways). Roads between municipalities that did not lead to the capital were considered transversal, which, in general, are secondary roads with less importance than the radial highways.

The information about the Bolivia-Brazil gas pipeline was provided by the Brazilian Gasbol Transporter (TBG) [32], and the present study only considered the north section of the pipeline that occurs within SP (from the municipality of Cosmópolis to the municipality of Castilho). Demographic, environmental, socioeconomic and geographical data on the municipalities were obtained from the Brazilian Institute of Geography and Statistics and the Foundation State System of Data Analysis. Information about the highways and duplication works was provided by the São Paulo Roads Department.

Information about the presence of prisons was obtained from the website of the Prison Administration Service of São Paulo. Information regarding the Tiete River was obtained from the Integrated System of Water Resources Management of São Paulo.

Independent variables representative of the climatic conditions of the municipalities were also considered. This information was obtained from the Integrated Center for Agrometeorological Information of the Secretariat of Agriculture and Supply of the State of São Paulo, which provides daily information on maximum temperature, minimum temperature and precipitation levels recorded by a network of meteorological stations located in municipalities representative of all microregions of SP. The following information was considered from 109 municipalities that had at least 10 years of monthly information documented within the study period (1997 to 2014): monthly averages of maximum and minimum daily temperatures; total monthly precipitation; and number of rainy days per month.

### Statistical analysis

Once climatic information was obtained, the following factors were calculated for each of the 109 municipalities across the entire study period: the average monthly maximum temperature; the average monthly minimum temperature; the average monthly precipitation level; and the average number of rainy days per month. These values were georeferenced to the centroids of the 109 municipalities. For each of these variables, semivariograms were constructed that modeled the spatial dependence of this data and provided the weights used in the statistical interpolations or kriging. This allowed the construction of thematic maps with information on these variables for the entire study area. These procedures were performed in the *geoR* package [33] from R software [34]. With these maps, interpolated values for these four climatic variables were obtained for each of the centroids of the 645 municipalities of SP using the nearest neighbor tool of the QGIS program [35].

The survival analysis method was used to evaluate the time between onset of surveillance and the outcome (vector, canine and human autochthonous case appearance). The probabilities of appearance of the vector, canine case, and human case were calculated at 2, 5, 10 and 15 years. Survival curves were constructed by the Kaplan-Meier method for the independent variables and compared by the log-rank test. The univariate and multiple Cox regression model was used to calculate the hazard ratio (HR) with 95% confidence intervals (CI).

In the construction of the multiple Cox regression model, the stepwise backward method was used, in which variables with a descriptive level (*P*) less than 0.20 in the univariate analysis were considered as candidates for the multiple model. In the final model, only variables with a descriptive level (*P*) less than 0.05 were considered. All analyses were performed in the statistical software SPSS for Windows v. 18, and the level of significance was 5%.

## Results

Of the 173 SP municipalities with a documented presence of the vector, four (2.4%) did not have information about the month of detection, only the year. Of the 84 municipalities that reported autochthonous cases of human VL and the 108 municipalities that reported autochthonous cases of canine VL, three (3.6%) and 51 (47.2%), respectively, only had information about the year of first notification. In these cases, June was assigned as the month of detection or notification (midpoint of the year).

The probability of survival in 2, 5, 10 and 15 years for the presence of the vector, human cases and autochthonous canine cases are presented in Table 1. In the context of this analysis, the survival probabilities for the three considered outcomes are understood, respectively, as the probabilities of a given municipality to not present the vector, the canine case, and the human case after a certain period of time.

**Table 1:** Probability of appearance of the vector, canine case, and the human case of VL in the municipalities of the State of São Paulo, 1997–2014

Outcome	Event^a^	Censorship^b^	Probability of survival (%)			
	*n* (%)	*n* (%)	2 years	5 years	10 years	15 years
Vector appearance *Lu. longipalpis*	173 (26.8)	472 (73.2)	97.8	94.7	87.8	78.4
Canine case of VL	108 (16.7)	537 (83.3)	99.5	96.7	90.9	84.7
Human case of VL	84 (13.0)	561 (87.0)	99.8	98.4	93.8	88.5

^a^Event: Municipalities that presented a case of appearance of the vector, canine case or human case

^b^Censorship: Municipalities that did not present any case of appearance of the vector, canine case or human case

The database with all information for each of the 645 municipalities of SP is also presented (Additional file 1: Table S1), and the database contains information about the three dependent variables related to the vector and the disease in dogs and humans, their respective mesoregions and microregions (Fig. 1), and the values of all covariables considered in the analysis. Regarding the 173 with reported vector presented, 108 municipalities with reported canine VL presence, and 84 municipalities with human VL presence, 148 municipalities (85.5%), 101 municipalities (93.5%), and 84 municipalities (100%), respectively, were located in the western region of SP (Fig. 1, Additional file 1: Table S1).

In Additional file 2: Table S2 the information provided by TBG [32] regarding the municipalities of SP where the northern section of the gas pipeline crosses, the construction starts dates and the respective classification of the simultaneous or non-simultaneous construction in relation to the vector presence and disease in dogs and humans is presented. The construction began in Cosmopolis (November 1997) and ended in Castilho on the border with Mato Grosso do Sul (February 1999).

The results of kriging of the climatic variable for SP in the period of study are presented in Additional file 3: Figure S1 (average monthly maximum temperatures), Additional file 4: Figure S2 (average monthly minimum temperatures) and Additional file 5: Figure S3 (average monthly precipitation levels). These maps show that the western part of SP is hottest and driest of SP. Moreover, many of municipalities that borders the states of Minas Gerais and Mato Grosso do Sul or are close to them are the hottest and driest in the western region of SP.

Table 2 presents the factors associated with the presence of the vector, the canine cases, and the human cases in the municipalities of SP. In relation to the adjusted results, the presence of the Marechal Rondon highway showed the highest positive association with the three factors, with minimum temperature being second and maximum temperature being third.

**Table 2:** Variables associated with the appearance of the vector, the canine cases, and the human cases of VL in the municipalities of São Paulo, 1997–2014

Variables	Non-adjusted hazard ratio (95% CI)	Adjusted hazard ratio (95% CI)	*P* ^b^
Vector appearance *Lu. longipalpis*			
Marechal Rondon highway^a^	36.69 (22.03–61.08)	20.00 (11.14–35.92)	<0.001
Minimum temperature	3.11 (2.59–3.75)	2.47 (1.94–3.16)	<0.001
Transverse highways^a^	3.04 (2.16–4.28)	2.16 (1.47–3.16)	<0.001
Maximum temperature	2.01 (1.76–2.29)	1.28 (1.08–1.51)	0.004
Canine cases of VL			
Marechal Rondon highway^a^	51.95 (30.87–87.41)	17.16 (8.06–36.54)	<0.001
Mato Grosso do Sul border^a^	7.50 (3.64–15.45)	3.39 (1.49–7.68)	0.004
Minimum temperature	3.96 (3.03–5.18)	2.48 (1.56–3.95)	<0.001
Maximum temperature	2.41 (1.98–2.92)	3.08 (1.96–4.83)	<0.001
Presence of a prison^a^	2.07 (1.33–3.24)	1.87 (1.07–3.26)	0.028
Microregion headquarters^a^	1.81 (1.08–3.04)	2.20 (1.20–4.03)	0.011
Tietê River^a^	1.65 (1.13–2.40)	2.90 (1.70–4.93)	<0.001
Precipitation	0.99 (0.99–1.00)	1.01 (1.00–1.01)	<0.001
Rainy days	0.94 (0.93–0.96)	0.98 (0.96–1.00)	0.049
Human cases of VL			
Marechal Rondon highway^a^	40.78 (24.54–67.78)	13.47 (7.23–25.10)	<0.001
Minimum temperature	4.91 (3.48–6.91)	2.70 (1.61–4.55)	<0.001
Presence of a prison^a^	2.83 (1.76–4.54)	3.82 (2.28–6.40)	<0.001
Maximum temperature	2.61 (2.06–3.30)	2.22 (1.34–3.68)	0.002
Precipitation	0.99 (0.99–1.00)	1.01 (1.00–1.01)	0.037
Rainy days	0.92 (0.91–0.94)	0.97 (0.94–1.00)	0.023

^a^Reference category: No

^b^Multiple adjusted Cox regression

*Abbreviation*: 95% CI, 95% confidence interval

**Fig. 2:**
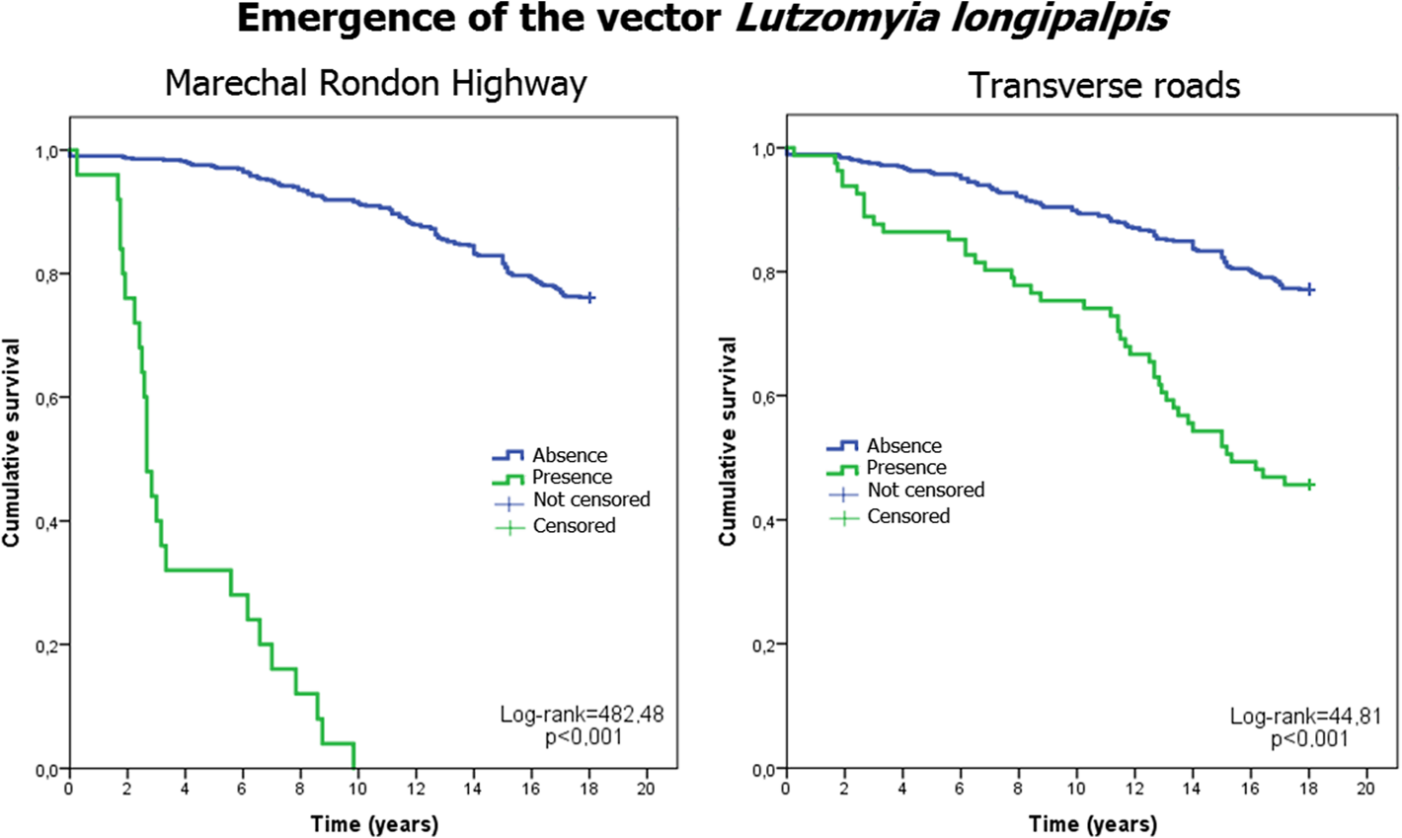
Survival curves and *Lu. longipalpis*. Emergence of *Lu. longipalpis* and the two associated categorical variables (the Marechal Rondon highway and transverse highways) from 1997 to 2014. Data for the municipalities of São Paulo, Brazil

**Fig. 3:**
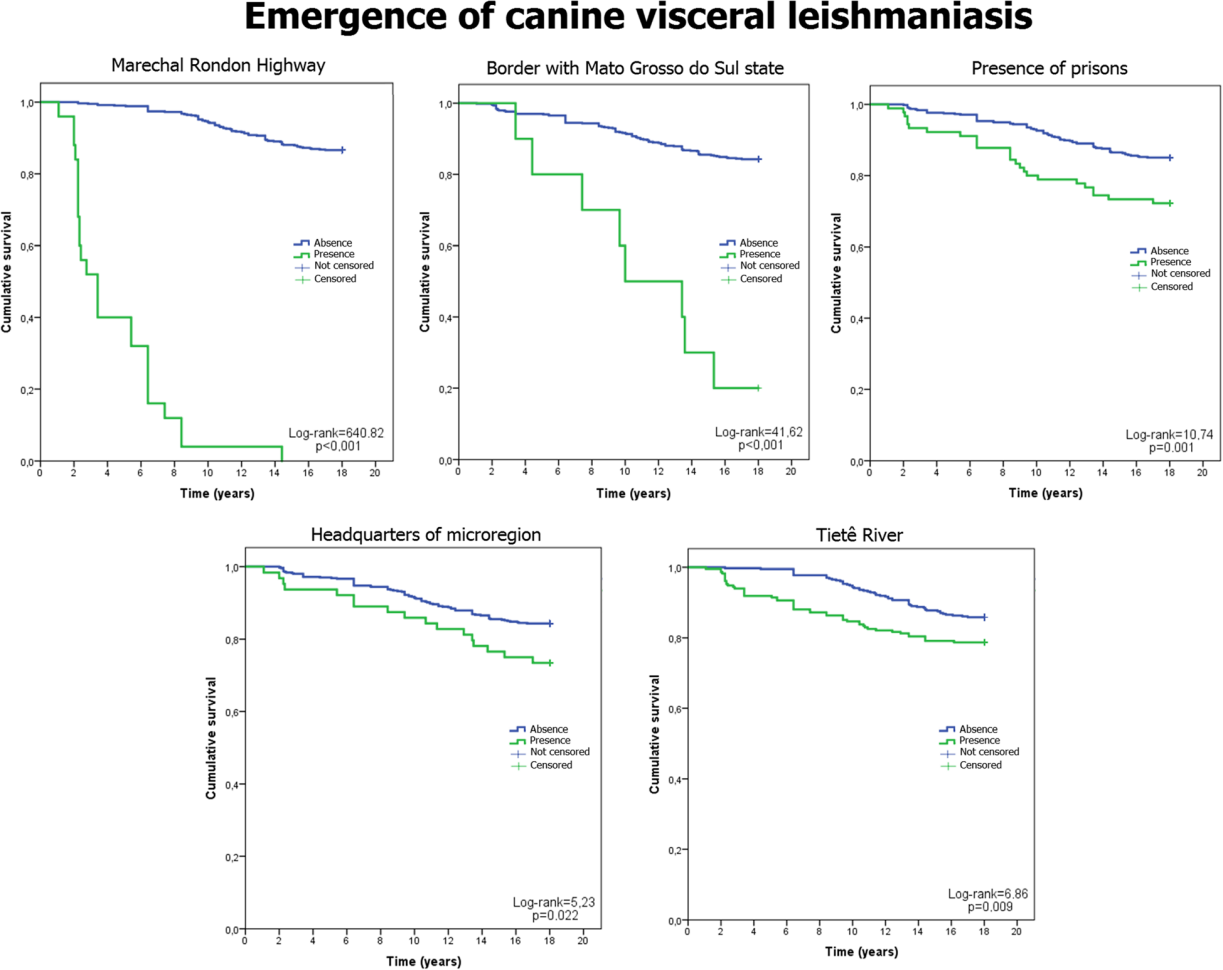
Survival curves and canine cases of VL. Emergence of canine cases of VL and the five associated categorical variables (the Marechal Rondon highway, Mato Grosso do Sul border, presence of a prison, microregion headquarters and the Tietê River) from 1998 to 2014. Data for the municipalities of São Paulo, Brazil

**Fig. 4:**
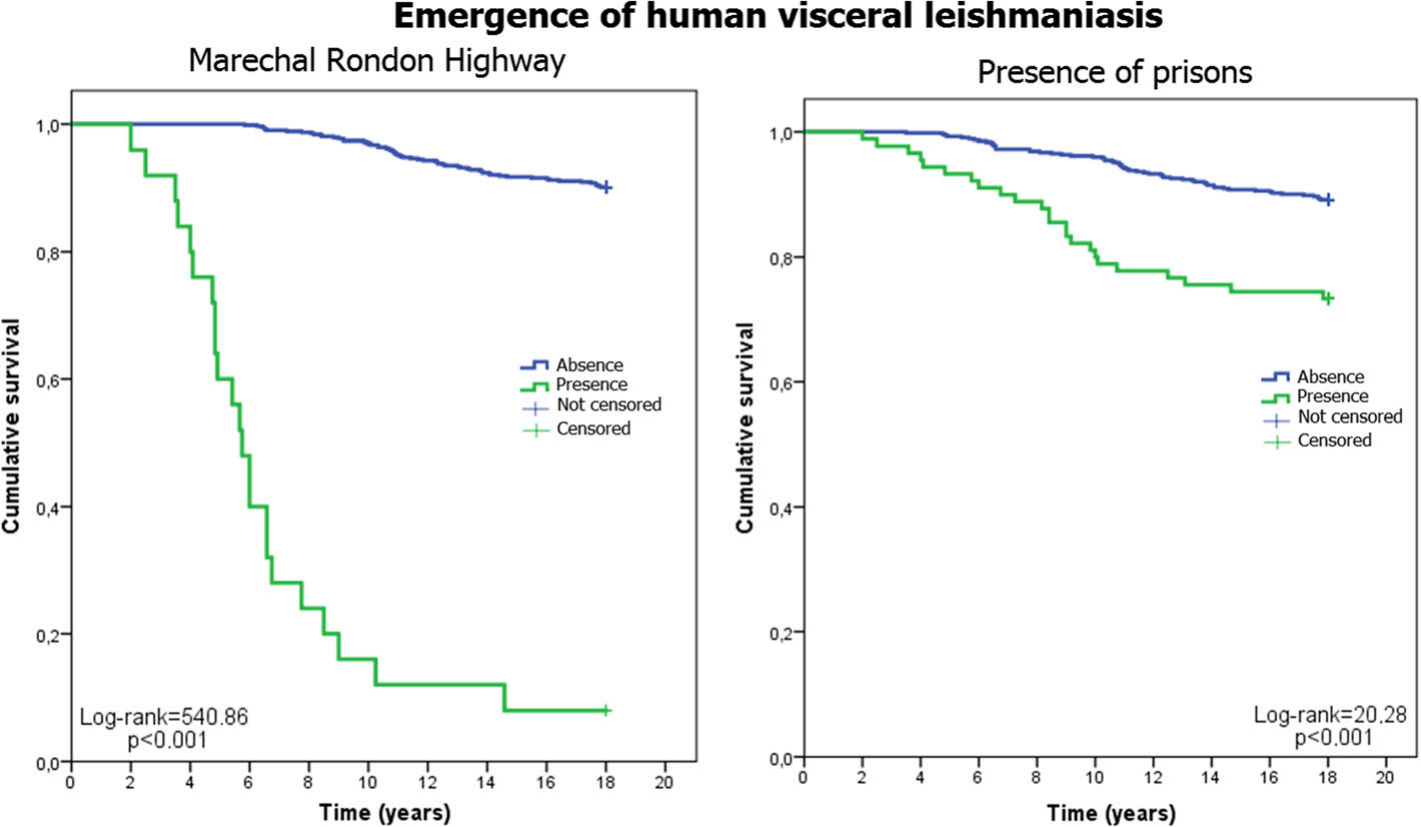
Survival curves and human cases of VL. Emergence of human cases of VL and the two associated categorical variables (the Marechal Rondon highway and presence of a prison) from 1999 to 2014. Data for the municipalities of São Paulo, Brazil

The presence of transverse highways was positively associated with the presence of the vector; the Mato Grosso do Sul border, the presence of a prison, the microregion headquarters, and the presence of the Tietê River were positively associated with the presence of canine cases; however, only the presence of a prison was positively associated with the presence of a human cases. Precipitation was positively associated with the presence of canine and human cases in the municipalities, while the number of rainy days was negatively associated with the presence of canine and human cases. Figures 2, 3 and 4 show the survival curves for the detection of the vector, canine cases, and human cases, respectively, and each of the categorical independent variables associated in the Cox regression model.

## Discussion

### Association with highways

The present study, using the survival analysis technique, showed that the presence of the Marechal Rondon highway was the variable most strongly associated with the vector presence and occurrence of canine and human VL cases in the municipalities of SP. Similarly, other studies have indicated that both vector dispersion and VL expansion in SP occurred along the route of this highway [10, 16, 20, 21]. Sevá et al. [15] statistically tested this hypothesis, but found a significant association only between the presence of canine VL cases in a given municipality and its distance to the Marechal Rondon highway. This association may be related to the fact that this highway is a channel that facilitates the transportation and migration of people and animals, including individuals and animals who are seropositive or have VL [9, 11, 13, 14, 17]. In Brazil, the migratory flow and circulation of people and animals occurs predominantly highway travel, thus highways play an important role in the spread of diseases [12, 13].

In addition to the presence of the Marechal Rondon highway, the dispersion of the vector was associated with the entire network of transverse highways in SP; however, this association was not observed for the dispersion of canine and human VL cases. A study conducted in the SP region of São José do Rio Preto revealed that the dispersion of *Lu. longipalpis* has been through neighborhood between municipalities that are usually connected by neighboring roads, which are the most frequent type of transverse highways in SP [18]. This observation is most likely explained by the mechanisms through which the vector is spread. One potential method of dispersion is through the transport of contaminated materials, such as soil, grass and chicken feces, across small to medium distances [6]. Another way would be the dispersion of adult *Lu. longipalpis* through motor vehicles [12].

### Association with the states that border SP

The present study identified the border of Mato Grosso do Sul as one of the factors associated with the expansion of canine VL in SP and this association may also be related to the fact that the Marechal Rondon highway is a continuation of BR-262 in SP. Even though the border with this state was not associated with the dispersion of *Lu. longipalpis* and the expansion of human VL in SP, several studies have indicated this state as the origin of VL in SP [10, 15, 16, 21, 22, 36].

Casanova et al. [36] found that *Lu. longipalpis* from Araçatuba, located in the western part of SP, produces the (S)-9- methylgermacrene-B pheromone and *Lu. longipalpis* from Espirito Santo do Pinhal, in the eastearn part of SP, produces the cembrene-1 pheromone. In a more recent study, Casanova et al. [10] found that the (S)-9-methylgermacrene-B population of this vector is widely distributed in the western part of SP and the cembrene-1 population is restricted to the eastern part of SP. They also showed that the expansion of VL in SP has followed only the dispersion of the (S)-9-methylgermacrene-B population.

It is worth noting that Bray et al. [37, 38] showed that the pheromone of *Lu. longipalpis* from Campo Grande, the capital of Mato Grosso do Sul, was also characterized as (S)-9-methylgermacrene-B. Motoie et al. [22] studied the genetic characteristics of *Leishmania infantum* from SP using multilocus microsatellite typing and concluded that this analysis is in agreement with the hypothesis of the introduction of CVL in the western region of SP be due to the movement of dogs and humans from the State of Mato Grosso do Sul.

### Association with gas pipeline construction

A primary motivation of the present study was to test the hypothesis proposed by Cardim et al. [16], who based their hypothesis on the study by Correa-Antonialli et al. [11], that the construction of the gas pipeline contributed to the dispersion of *Lu. longipalpis* and expansion of canine and human VL cases in SP. Sevá et al. [15] tested this hypothesis and identified a statistically significant relationship between the vector presence in a municipality and its distance to the gas pipeline, a marginally significant relationship between this distance and the human VL presence, and no significant relationship between the gas pipeline and canine VL presence; the authors of this study considered these results to be inconsistent [15].

A possible explanation for this inconsistency could be the high collinearity between the distance of the municipalities to the gas pipeline and to the Marechal Rondon highway, which both have similar paths and are spatially close [32]. In the present study, this situation was avoided by considering the presence of the highway and not its distance and by not only considering the presence of the gas pipeline in the municipality, but also assuming that the gas pipeline could influence the vector dispersion and expansion of the VL only during the period of construction development or within one year of its completion.

The model of the present study, in contrast to the results of previous studies [11, 15, 16], did not detect any relationship between the gas pipeline and the vector dispersion and VL expansion, which could be related to the differences in the construction of the Bolivia-Brazil gas pipeline in Mato Grosso do Sul and the SP states. For the gas pipeline constructed in Mato Grosso do Sul, the construction occurred between 1998 and 1999 and the work moved west-to-east, which followed the same trajectory of the expansion of the human VL cases [11]. For the gas pipeline constructed in SP, the construction of the northern section started in 1997 in the Cosmopolis, Campinas region, and was completed in 1999 in Castilho [32], which advanced east-to-west and was therefore inversely related to the vector dispersion path and VL expansion trajectory in SP (Additional file 2: Table S2) [10, 15, 16, 20].

Comparisons between the gas pipeline construction and the vector dispersion and VL expansion in SP suggest that the results of the present study are plausible. In the mesoregion of Araçatuba, the construction of the gas pipeline began in July 1998 and the vector had already been detected in Araçatuba for more than a year (April 1997). A similar situation occurred in the Andradina microregion. The first autochthonous canine case in Araçatuba had already been detected six months prior to the start of the gas pipeline construction (February 1998) (Additional file 2: Table S2). Regarding the association between the gas pipeline construction and the expansion of the human VL cases, only two municipalities (Araçatuba and Birigui) reported the presence of cases during the gas pipeline construction period or within one year of its completion (Additional file 2: Table S2). In principle, these results weaken the association between the construction of the gas pipeline and the spread of the vector and disease; however, the results do not rule out the possibility. Additional studies are needed to better evaluate this hypothesis.

### Association with presence of prisons

The association between the presence of municipality prisons and the appearance of autochthonous canine and human VL cases emphasizes the role of the people and animal migration in the dispersion of VL [9, 11–14]. In 2013, Brazil had more than 500,000 prisoners, with approximately 40% of this total population residing in SP and, in order to deal with this surplus demand, the State decided at the end of the 1990’s to build large prisons in the interior of the State. From then on, especially between 1998 and the middle of the following decade, the number of prisons in SP increased considerably, so that most of the prison units in Brazil are now located in SP [25]. This growth occurred in small and medium-sized cities, especially in the western region of SP [24]. Since the prisoners serve their sentences in municipalities different from their origins, the families of the prisoners often move to visit them mainly on the weekends, constituting themselves to floating populations. In addition, if these relatives choose to stay in the municipality, some of these populations may be characterized by temporary migrants [24, 25].

Given the presented information, specifically the period of greatest increase in the number of prisons (late 1990’s and mid-2000’s) and the region with the largest number of new prisons (western region of SP), the association between the presence of prisons and canine and human VL case dispersion observed in this study is supported. In our study this variable “presence of prisons” should be understood as indicative of migration and flow of people, which is a factor known to be responsible for the increase in the number of VL cases [9, 11–14]. In addition, one can infer that these population movements also include animals involved in the transmission cycle, and that these populations tend to settle in precarious places that are propitious for vector development and occurrence of VL [14, 39]. However, this hypothesis, first raised by the present study, should be further investigated through future studies to better evaluate its pertinence.

### Association with environmental variables

The positive association between mean maximum and minimum annual temperatures and the presence of the vector in the municipalities reflects the fact that most of municipalities are located in the western region of SP, which is characterized by the highest maximum and minimum temperatures in the state [40]. This result is consistent with other studies [14, 15, 26, 41]. Regarding rainfall, some studies have indicated that there is an increase in vector density during the rainy season [28, 41–43], and other studies have shown an increase in density after the rainy season or after peak rainfall [44–47]. This association could be explained by the hypothesis raised by Rutledge & Ellenwood [48] that moderate levels of rain during the rainy season increases the density of sandflies, but that excess rainfall (with flooding of soil and destruction of breeding sites) would decrease the density of sandflies. The lack of association between precipitation and vector presence in the present study could be attributed to the fact that the study did not consider the density of the vector, but rather its presence.

In relation to the positive associations between temperature and occurrence of canine VL cases, which was also detected by Sevá et al. [15], and human VL cases, which was also detected by Almeida et al. [26], these could be understood as a consequence of the association of the vector with this variable. The positive association between canine and human VL cases with rainfall and the negative association with rainy days could also be a reflection of factors related to vector density.

A question that could be raised is whether the association of Marechal Rondon highway with the dispersion of the vector and expansion of VL is SP would be a result dependent on environmental variables. First, this result was adjusted by climatic factors (maximum and minimum temperature and precipitation level). Also, the hottest and driest part of SP borders the states of Minas Gerais and Mato Grosso do Sul. Secondly, other highways crossing the western region of SP from these states, and not only Marechal Rondon, could be a vehicle for the entrance and dispersion of the vector and the disease in SP.

### Association with other variables

Among the covariates considered in the analysis, the MHDI, composed by longevity, education and income, represents the living conditions of the residents of the municipalities, a factor known to be associated with the occurrence of VL. However, the results of the present study indicated no correlation between MHDI and vector presence or canine and human cases, and this is partially supported by the results obtained by Sevá et al. [15]. In contrast, Oliveira et al. [20] showed that, in the period from 1998 to 2014, the headquarters of microregions played an important role in the expansion of canine and human VL cases because approximately 75% of the autochthonous cases were first detected in these regions. The reasons for this association could be attributed to the fact that these regions are considered to be economic, educational and health centers, which attract a greater movement of people. The association of the Tietê River with the occurrence of canine VL cases would also fit into this category because the river is a travel route and leisure area for a large number of people.

The methodological highlight of the present study was the use of survival analysis, a statistical tool often used in observational oncology and cohort studies, but having not documented use in literature for a study of this kind. Survival analysis can be used to evaluate the relationship of a variable of interest or dependent variable and a given event in relation to a certain time period or occurrence [49]. Among the various methodological options available, the Kaplan-Meier method was selected for this study because it does not use fixed time intervals, but rather is determined by the occurrence of the event, and the Cox regression model was used because there was interest in studying the impact of some risk factors during the time until the occurrence of the event of interest [49].

### Limitations

The present study has limitations. First the study used secondary data regarding the date of vector detection in the municipality or of canine and human autochthonous VL cases. The date of vector detection was biased by operational issues because collections were not conducted throughout the year. For the detection of canine cases, there was uncertainty regarding the month of confirmation of the first autochthonous case in a large number of municipalities. As for the detection of the human cases, problems related to underreporting may have delayed the detection of the first autochthonous cases. As these limitations are more related to the month of occurrence of these phenomena, the early detection systems of the vector and the diseases in dogs have good sensitivities [20]. Secondly, human VL is a compulsory notification disease; therefore, it can be affirmed that there was precision in relation to the years of occurrence of the studied phenomena. The fact we did not take into account information about the presence of *Lu. longipalpis* and the occurrence of CVL and HVL in the municipalities of the states that border with SP is also a limitation of our study.

## Conclusions

The presence of the Marechal Rondon highway in the municipalities was the factor most strongly associated with the dispersion of *Lu. longipalpis* and the expansion of canine and human VL cases in SP. The average mean maximum and minimum monthly temperatures were also positively associated with the three phenomena. The presence of transverse highways was positively associated with the presence of the vector; the Mato Grosso do Sul border, the presence of a prison, the microregion headquarters, and the presence of the Tietê River were positively associated with the presence of canine cases; and the presence of some prisons was positively associated with the presence of human cases. The construction of the Bolivia-Brazil gas pipeline in the municipalities was not associated with the three events studied. To the best of our knowledge, this is the first study to identify an association between the presence of a prison and vector and VL dispersion, which is believed to be attributed to the temporary migration of the families of the prisoners; however, future studies are needed to further elucidate this relationship. In addition, the use of survival analysis in this study was critical to the identification of factors associated with vector dispersion and disease spread. These results can be useful in the improvement of surveillance and control activities of VL.

## Additional files

Additional file 1: **Table S1.** Information about all variables for each municipality used in this study. (XLSX 110 kb)

Additional file 2: **Table S2.** Information about gas pipeline. Municipalities of SP where the northern section of the gas pipeline crosses, the construction starts dates, and the respective classification of the simultaneous or non-simultaneous construction in relation to the vector presence and disease in dogs and humans are presented. (XLSX 14 kb)

Additional file 3: **Figure S1.** Average monthly maximum temperatures, State of São Paulo, 1997 to 2014. (PNG 412 kb)

Additional file 4: **Figure S2.** Average monthly minimum temperatures, State of São Paulo, 1997 to 2014. (PNG 432 kb)

Additional file 5: **Figure S3.** Average monthly precipitation levels, State of São Paulo, 1997 to 2014. (PNG 505 kb)

## Abbreviations

CI: Confidence interval
MHDI: Municipal Human Development Index
SP: State of São Paulo
TBG: Brazilian Gasbol Transporter
VL: Visceral leishmaniasis
