# Supplementary figures and images for: Dispersion of Lutzomyia longipalpis and expansion of visceral leishmaniasis in São Paulo State, Brazil: identification of associated factors through survival analysis
Source: Parasit Vectors. 2018 Sep 10;11:503. doi: 10.1186/s13071-018-3084-1 (PMC6131759; doi:10.1186/s13071-018-3084-1)

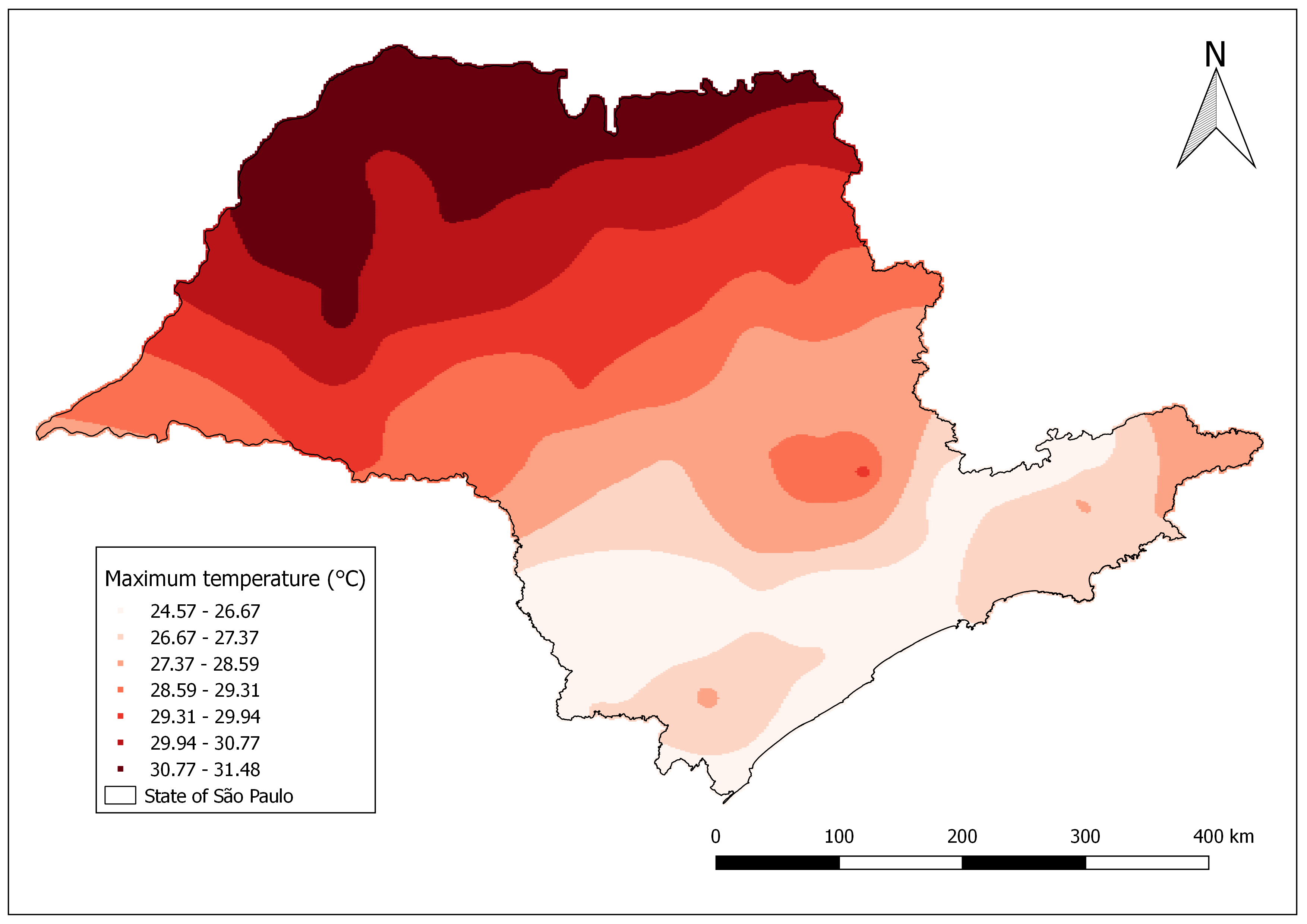

Supplement: Supplementary file 3 — Figure S1. Average monthly maximum temperatures, State of São Paulo, 1997 to 2014. (PNG 412 kb) [file 13071_2018_3084_MOESM3_ESM.png]

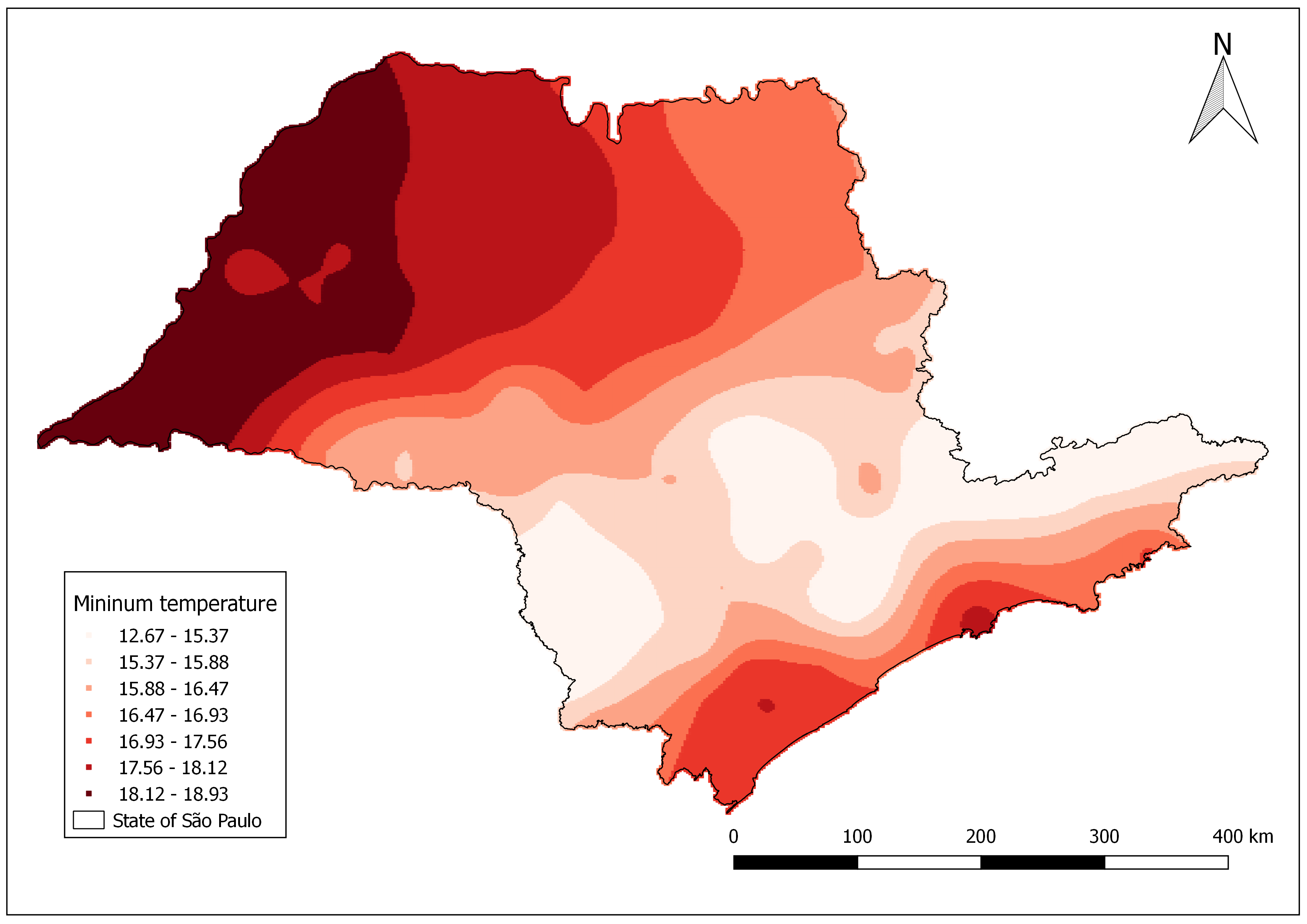

Supplement: Supplementary file 4 — Figure S2. Average monthly minimum temperatures, State of São Paulo, 1997 to 2014. (PNG 432 kb) [file 13071_2018_3084_MOESM4_ESM.png]

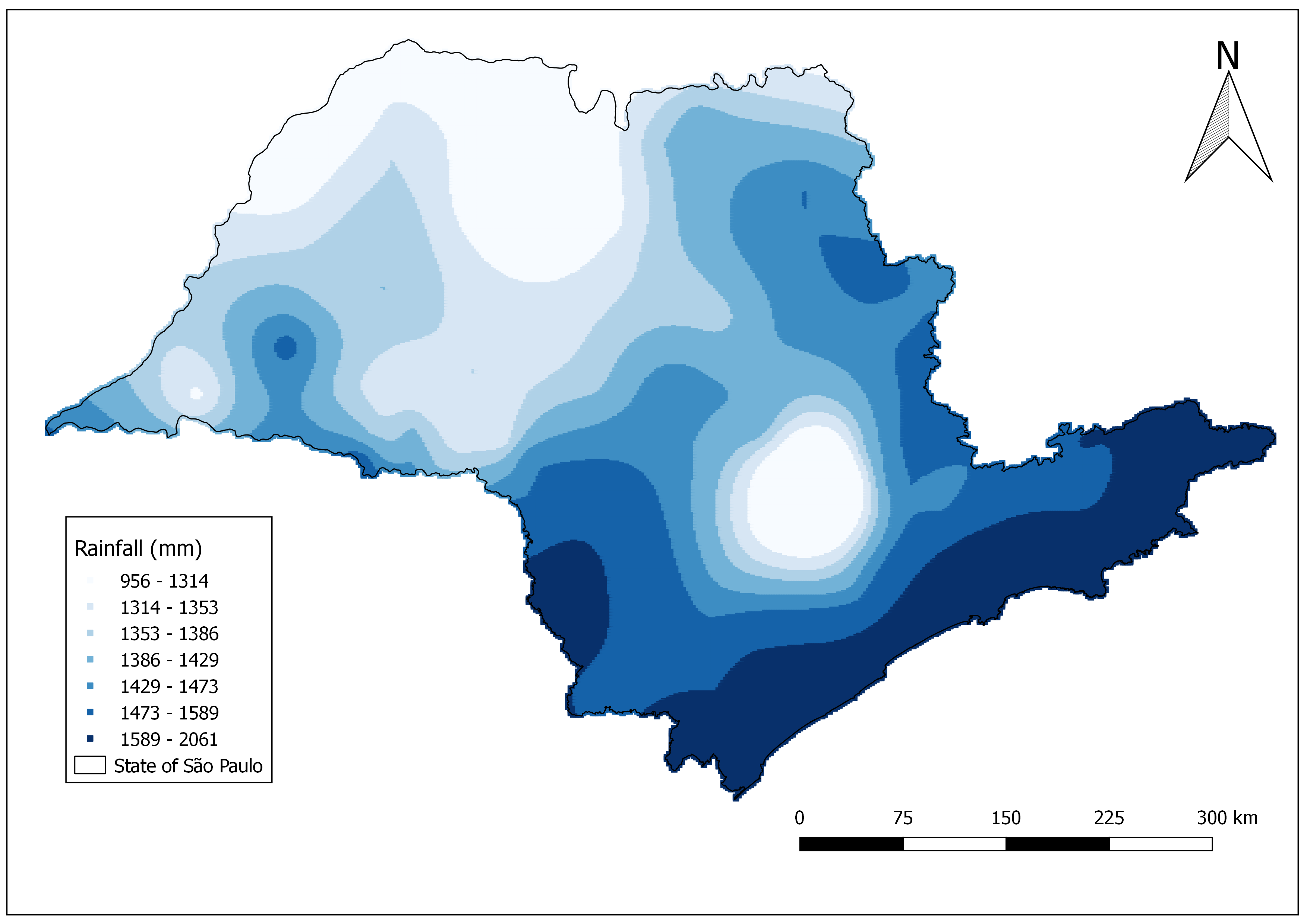

Supplement: Supplementary file 5 — Figure S3. Average monthly precipitation levels, State of São Paulo, 1997 to 2014. (PNG 505 kb) [file 13071_2018_3084_MOESM5_ESM.png]
